# Do You Want to Make a Battery? Insights from the Development and Evaluation of a Chemistry Public Engagement Activity

**DOI:** 10.1021/acs.jchemed.4c01123

**Published:** 2024-11-01

**Authors:** John O’Donoghue, Natalia García Doménech, Dearbhla Tully, Niamh McGoldrick, Fiona McArdle, Mary Connolly, David J. Otway, Will Daly, Lynette Keeney, Mervyn Horgan

**Affiliations:** † School of Chemistry, Trinity College Dublin, Dublin, Ireland D02 P3X2; ‡ School of Life Sciences, Atlantic Technological University, Sligo, Ireland F91 YW50; § School of Chemistry, University College Cork, Cork, Ireland T12 K8AF; ∥ Tyndall National Institute, University College Cork, Cork, Ireland T12 R5CP; ⊥ Lifetime Lab, Old Cork Waterworks, Cork, Ireland T23 N828

**Keywords:** Electrochemistry, Public, Outreach, Researchers

## Abstract

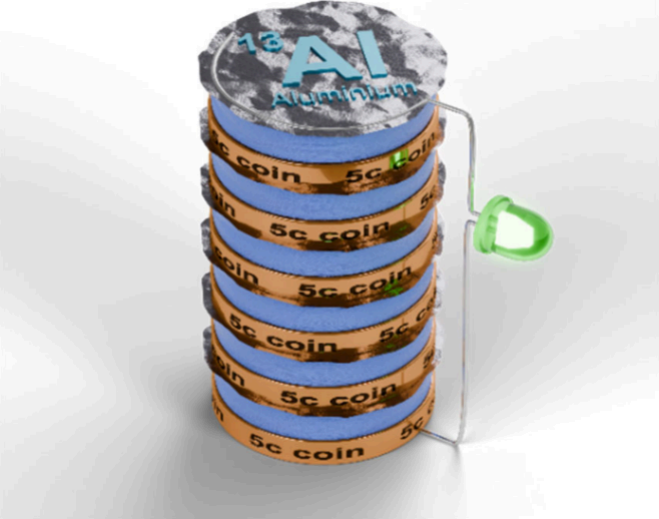

Chemistry is often associated with formal learning environments and has been described as overly serious by the general public, lacking some of the fun and energy of other sciences. However, it is difficult to provide hands-on chemistry activities outside the lab and other formal learning environments. Here, a simple electrochemistry based activity has been used for public engagement using household items and play dough to create a fun and playful experience for all ages. The benefits afforded by outdoor learning for developing curiosity and interest in science has also been explored through different event formats. The use of a “Smiley Stand” with “emojis” for gathering participant feedback was successfully deployed alongside interviews with the “Ambassadors” who facilitated the activity. Overall, it was found that the activity encouraged two-way conversations between the participants and the ambassadors, with few negative responses and many positive ones received. The activity also impacted the ambassadors’ own view of science.

## Introduction

A 2015 public attitudes to chemistry survey found that most people were unable to see chemistry as being personally relevant, associating it with school and the development of drugs and medicines. Chemistry was said to lack some of the fun and energy of other sciences; generally viewed as serious in personality, comprising difficult, repetitive experiments.^1^ A recent survey also found similar results, with chemistry mentioned alongside physics as a “difficult subject”.^2^ Using a “Draw-a-Scientist Test” (DAST), it has also been found that children mostly perceive scientists to be “*male, elderly or middle-aged, wearing a lab coat and glasses, on his own in a laboratory performing experiments with test tubes*”.^3^

It is desirable to break these stereotypes through the provision of accessible activities with relevant applications, run by diverse role models which are reflective of chemistry’s broad and inclusive community. This should be achieved alongside the use of inclusive environments, creating a sense of belonging and improving self-confidence.^4−9^ However, by its very nature, many hands-on chemistry activities require a formal setting, due to the chemicals, equipment, and reactions involved. Many “outreach” activities also take place in formal environments such as schools, universities and museums, which, despite best intentions, may contribute to the “serious” image.^10−12^ Similarly, PPE and formal environments are usually needed for “fun” chemistry demonstration shows.^13−16^

The environment in which learning takes place can have an impact, with outdoor learning having the potential to develop curiosity and interest in science subjects.^17,18^ Recently, the introduction of an “art” dimension into conventional science education activities has also shown to be beneficial.^19−21^ The acronym STEAM is used to define such interdisciplinary learning, defined as Science, Technology, Engineering, Art, and Maths. The authors have previously reported chemistry activities that are safe for all ages and applicable for use outside formal environments.^22^ The COVID pandemic also encouraged the development of “At-Home Experiments”, most of which retained well-established scientific processes but are nonetheless safe to perform outside formal environments.^23−26^

Away from drugs and medicine, a relevant chemistry application is the area of energy. Lithium ion batteries have become key to stabilizing the inconsistency of renewable energy like solar and wind and for use in electric vehicles (EVs).^27−30^ Electrochemistry underpins battery functionality, in addition to numerous analytical techniques.^31,32^ However, electrochemistry has also been identified as a difficult topic for chemistry learners due to misconceptions, misunderstandings and a perceived lack of relevance.^33−36^ In Ireland, it has been found that electrochemistry is one of the least attempted and most poorly answered topics on exams.^37^

In response, there have been numerous reports of interventions, but the majority are aimed at higher education students or secondary schools or both.^33−36,38−43^ A well-known and frequently used electrochemistry activity for “outreach” and education is colloquially known as “the lemon battery”. Based on the historic Daniell cell, galvanic cell, and voltaic pile, it involves two different metal electrodes from opposite sides of the electrochemical series inserted into a lemon which acts as the electrolyte. The chemistry and scientific pedagogy of this activity as an example of reduction/oxidation (redox) chemistry has been explored previously.^44,45^ However, there are no known reports of using these types of cells or similar for nonformal environments and/or public audiences.

## Activity Development

### Focus Group

A focus group with 21 science teachers from different schools was conducted in 2019 and were recruited at a student event through voluntary participation. When asked about ‘the chemistry of the lemon battery’, over half incorrectly stated that the critic acid was the source of electrons in the circuit. Only those with a chemistry and/or physics specializm were likely to correctly identify the metal anode. Therefore, it is proposed here that this activity may be associated with some misconceptions in relation to redox chemistry. The teachers noted that the confusion may arise from the fact that the battery is named after the electrolyte rather than the active metal.
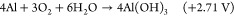
1Traditionally, zinc has been used as the anode for fruit/vegetable type batteries.^46−50^ More recently, the use of aluminum (Al) in metal-air batteries has attracted significant attention due to the theoretically high energy density compared to lithium ion batteries.^51−54^ Al is also affordable, recyclable, and widely available as a relatively abundant element. It is also accessible to public audiences since it is widely recognized as “kitchen foil”. Similar to zinc, the Al anode is oxidized while oxygen is reduced at a copper cathode (eq 1).^53^ Further details and half-cell equations are available in Supporting Information, Activity Testing.

### School Workshops

Subsequently, a nonformal school workshop was run with 160 secondary students in 5 schools. Using a “scientist in a classroom” model, early career science researchers (PhD and Postdoctoral), referred to here as “Ambassadors”, led the workshops.^11,12^ Students (15–18) were presented with the task of comparing different electrolytes for “voltaic piles” to investigate which was the most effective and why (Worksheets). Local context was also used within the narrative to help students appreciate the applications of battery chemistry in the real world.^55,56^ Feedback was gathered through anonymous online surveys sent to students (through their teacher who acted *in loco parentis*). All participants were also offered informed consent and told that the survey was voluntary and anonymous, which included the freedom to withdraw at any time.^57^

Overall, 89% of the student respondents (*n* = 26) found the activity interesting or very interesting with one student stating that “*it was a very fun activity*”. Through an open-ended question, they also expressed that they “*learned about cells*”, “*the difference between a battery and a cell*” and “*how batteries work*” for the most part. The teachers (*n* = 5) acknowledged that the chemistry concepts were correctly suited to upper secondary school. However, they felt the activity was better suited to younger audiences, due to the emphasis on “arts and crafts” skills rather than “traditional lab skills” like handling glassware.

### Public Engagement

**Figure 1:**
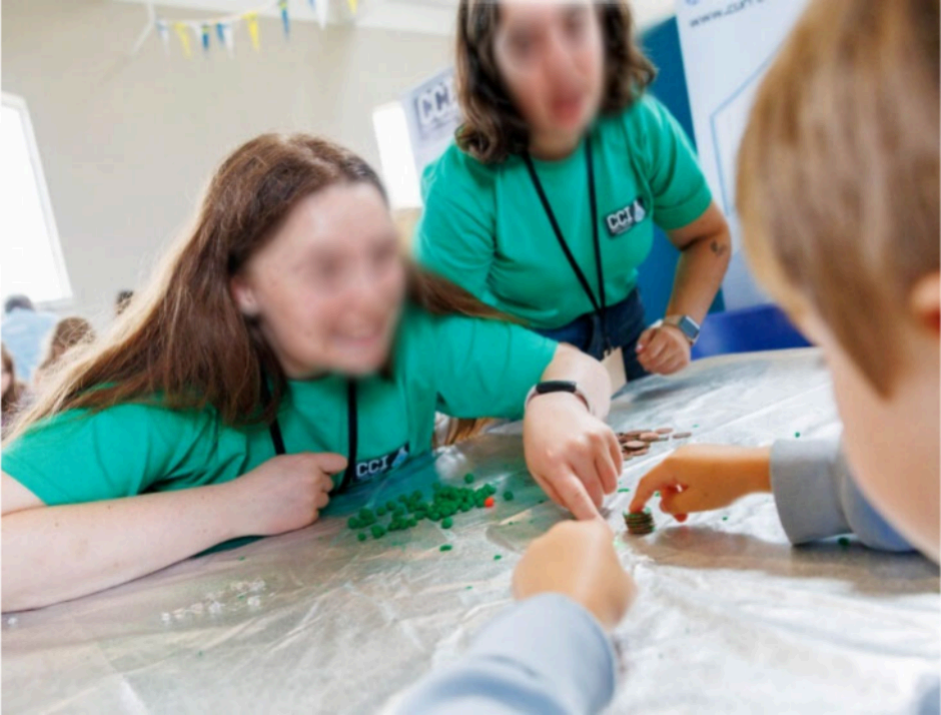
Ambassadors facilitating the activity at a public engagement event.

The activity was then adapted for the public using a “microworkshop” format, involving participants briefly sitting with Ambassadors at tables and chairs (Figure 1). To create an enjoyable and fun experience, a “Lead Ambassador” maintained a low number of participants per Ambassador to about 5:1. The Lead Ambassador acts as a “front of house” host like a Maître d’ in a restaurant, controlling the crowds, and assigning participants to tables as space becomes available. It is proposed here that this format encourages two-way conversion with the Ambassadors, who act as tangible scientific role-models in a fun, open and accessible environment.

Due to the rapid turnaround in participants, the aluminum disks and electrolyte spacers are precut, so participants only need to assemble the voltaic pile. The Ambassador demonstrates how to construct the first cell, leaving the participants to make the others. Although the LED could be lit with four cells using salt-water as the electrolyte (Activity Testing), more cells were employed to reduce the possibility of a “failed experiment” which may have detrimental effects on a participant’s confidence.

However, the corrosive salt-water had a detrimental effect on the activity over time. Recently, the use of “gel polymer electrolytes” for Al–air batteries has attracted significant attention and it is claimed that their success is due to the polymer matrix imbibing various liquids and molecules through swelling.^53,58^ Playdough (commercial name Play-Doh) is a well-known, nontoxic, reusable “modeling clay” or “putty”, commonly used for “arts and crafts”. It is commercially available or can be homemade from corn starch, salt, water, and vegetable oil. Starch is a mixture of two polysaccharides, amylose (linear) and amylopectin (branched). Hot water releases the amylose from the starch granule, causing it to swell and gelatinize, while amylopectin is only soluble in cold water.^59,60^

**Figure 2:**
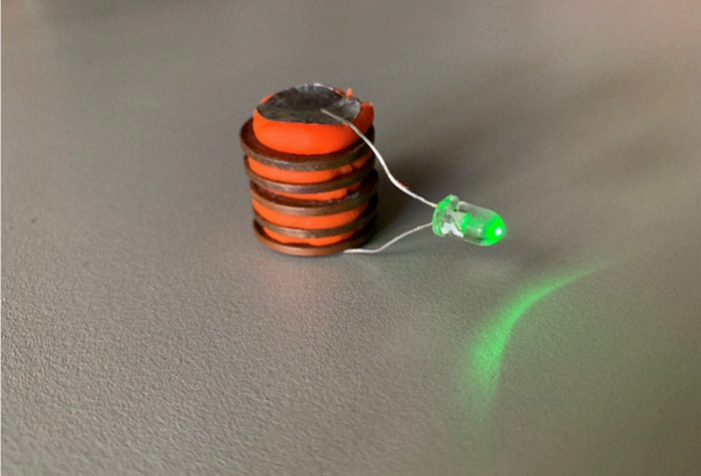
Five-cell voltaic pile with a functioning LED made from cells of aluminum foil, Playdough (orange), and copper coated coins stacked together.

It is proposed here that playdough functions as an electrolyte in 3 ways. First, the amylose acts as an intrinsically conducting polymer (ICP) due to its tightly packed linear helical conformation, inside of which ions can flow.^61,62^ Second, it provides a stable pH which is favorable for the anode reaction.^53^ Finally, it can retain salt within its matrix to increase its conductivity. Substituting for the cloth and salt-water, the playdough eliminated the corrosion, provided structural rigidity, and gave similar voltages (Activity Testing). Also, the playdough was instantly recognized by all participants and created an “arts and craft” based, playful atmosphere (Figure 2).

## Evaluation

**Figure 3:**
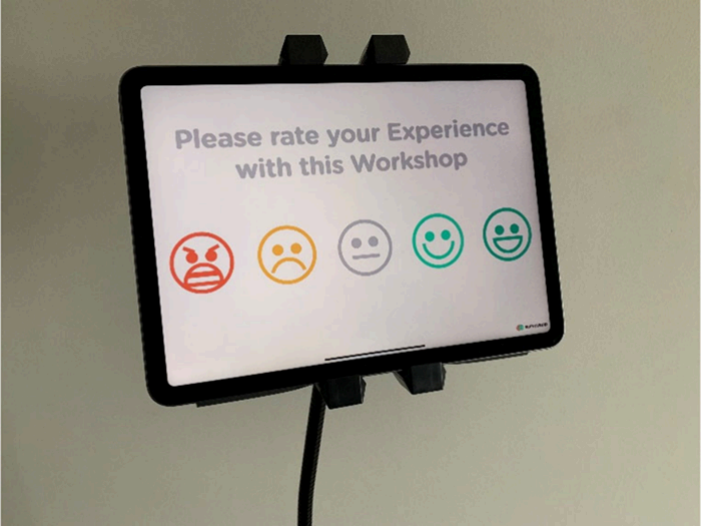
“Smiley Stand” used for gathering feedback at public engagement events described herein.

As well as aiding activity design, formal spaces can also facilitate feedback gathering through designated spaces, predictable timings, and contact details for survey distribution.^8,11,12,63^ For public spaces, the feedback instrument needs to be greatly simplified to accommodate the rapid turnaround, large numbers, and flexible timings.^64^ In the interest of sustainability and accuracy, a digital survey instrument was desired as well as real-time data to implement changes quickly. “Smiley Stands” using “emojis” are common in airports, supermarkets, and other retailers (Evaluation Data). They usually consist of color-coded buttons representing angry, neutral, or smiling faces (Figure 3).

In the interest of accessibility and to accommodate the rapid turnaround, a single question was asked in line with retail examples: “*Please rate your experience with this workshop*”. The color coded smiley faces ranged from “I hated it” (red-1), “I did not like it” (orange-2), neutral (gray-3), “I Liked it” (blue/green -4), and “I loved it” (dark green-5). Labels were tested at one event, but no difference was found compared to using the “smiley faces” without labels. In the interest of reducing language barriers, it was decided to use the icons without labels. The “Smiley Stand” was prominently located at the exit to the marquee, stand or room in which the activity was taking place, away from the Ambassadors to provide anonymity.

**Figure 4:**
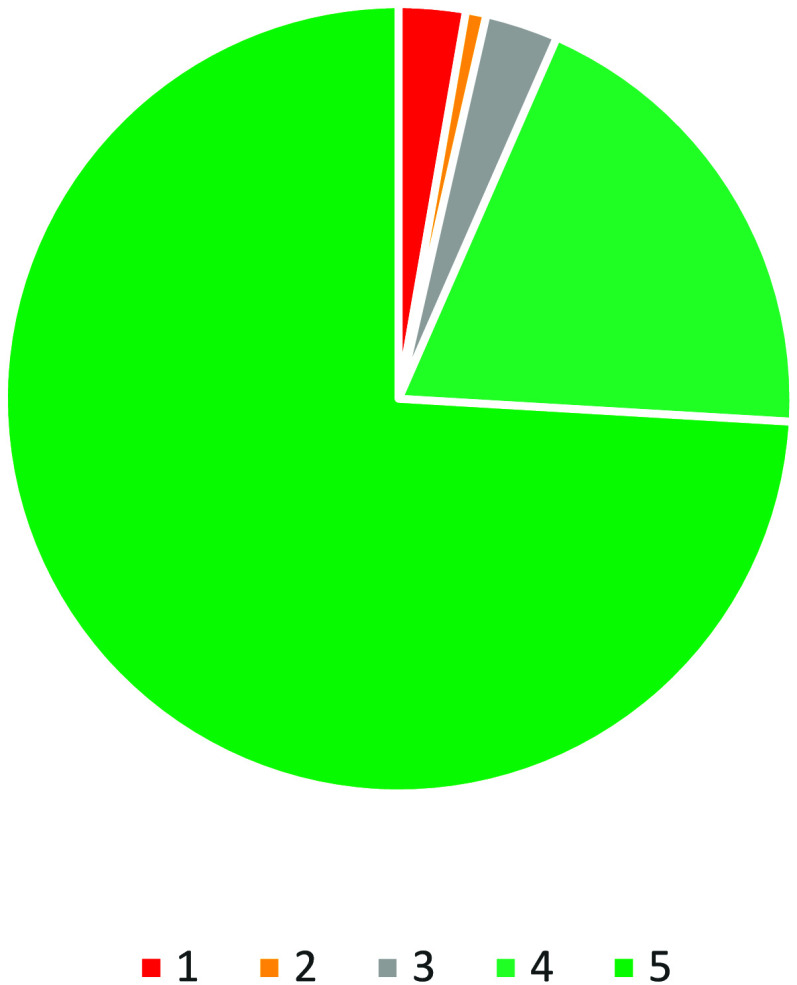
Total participant feedback received from the “Smiley Stand” from public engagement events (*n* = 4277).

In accordance with established ethical guidelines, participants were informed that taking part in the survey was voluntary and anonymous. A physical handout was provided with details about the activity and a link to a Web site containing more information.^57^ The data here represents about a third of the participants who completed the activity. Overall, the feedback from n = 4277 participants across 10 different events was overwhelmingly positive (Evaluation Data), with 74% of participants choosing the “I loved it” (5) response and a further 19% choosing the “I liked it” (4) response (Figure 4). Negative responses were consistently low with only 3% choosing the “I hated it” (1) response and 1% choosing the “I did not like it” (2) response, while the remaining 3% chose “neutral” (3).

Customer Satisfaction Score (CSAT) is a common metric for commercial Likert based feedback surveys. It is calculated by combining the highest two categories (4 and 5) and dividing by the total number of respondents. The result is expressed as a percent with 100% being the best and 0% being the worst. The overall CSAT score achieved here is an impressive 93%, far above the 75–85% normally considered excellent.^65^ No noticeable difference was found between events using the salt-water electrolyte and those that used playdough. This is most likely due to the efforts of the ambassadors to mitigate negative experiences through cleaning.

Although the instrument does not provide detailed responses, the data can help compare the activity for location type, event type and time slots. When running the activity at events that were based entirely indoors (*n* = 1761) a CSAT score of 91% was achieved, compared to 94% for events that were run entirely outdoors (*n* = 1603). As well as this, two distinct event categories emerged when comparing the data: science themed events (STEs) and mixed themed events (MTEs).

### Science Themed Events (STEs)

This category represents *n* = 2925 responses (68% of the total) and consists of 5 events; 2 primary science fairs and 3 science festivals. All STEs focused entirely on science, were free to attend, and offered a variety of science-based activities and demonstration shows. The 2022 science festival took place indoors over 1 day in the winter, while the 2023 and 2024 science festivals both took place outdoors over 2 days in the summer. Participants mainly consisted of children taking part in the activity with their parents/guardians or other adults. Both primary science fairs took place indoors over 4 days in the spring. Participants are primary school students (8–12 years) presenting their projects, with different schools attending each day. The projects are run alongside many different science activities and demonstration shows, similar to the science festivals. However, in contrast to the science festivals, the students took part in the activity with their classmates i.e. without an adult present.

**Figure 5:**
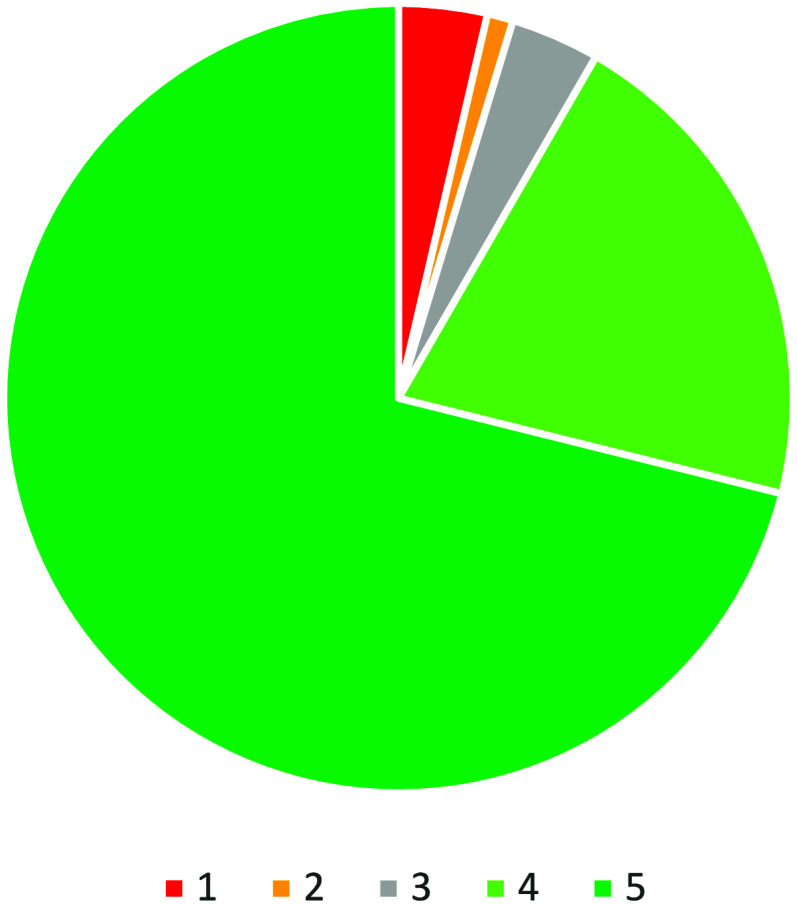
Breakdown of the feedback received for the activity at science themed events (*n* = 2925).

All the events in this category share similar feedback with the “I hated it” (1) response ranging from 2% for the 2024 science festival to 6% for the 2024 primary science fair, with all the others at 3%. The “I loved it” (5) response also varied significantly from 66% for the 2024 primary science fair, to 78% for the 2023 primary science fair (Figure 5). The CSAT score for STE events averaged 92% overall, which is slightly below the overall CSAT score of 93% and significantly below the CSAT score for MTEs (Evaluation Data).

**Figure 6:**
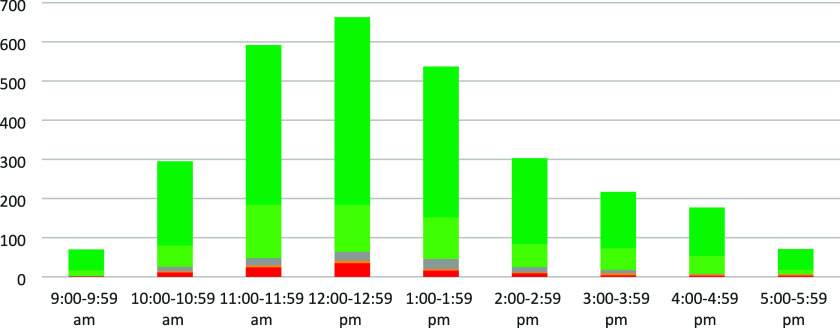
Breakdown of the responses received by hour for the activity at science themed events (*n* = 2925).

Because all these events comprised of full days, the responses can also be compared on an hourly basis (Figure 6). The highest number of negative responses were usually associated with busy periods such as lunchtime, in line with the highest number of responses overall. The CSAT score for the morning period (9–11.59 am) overall is 92%, dropping slightly to 91% for the lunchtime period (12–2.59 pm) and increasing again to 93% for the afternoon (3–5.59 pm). It is proposed here that the shorter turnaround between participants and the higher number of participants per Ambassador during busy periods (about 6:1) leads to more negative responses.

### Mixed Themed Events (MTEs)

The MTEs also consisted of 5 events: a 3 day outdoor agricultural show in the autumn of 2022, a 1 day outdoor food festival and half-day outdoor arts festival in the summer of 2024, and a mixed indoor/outdoor STEAM festival in the autumn of 2023 (1 day) and 2024 (2 days). Unlike the STEs, the activity here was offered among many nonscience activities. Participants mostly consisted of children taking part with their parents/guardians or other adults, like the STE science festivals. The agricultural show is the only event that required a cost for participants to attend and is also the only event where the participants consisted of teenagers taking part with their friends i.e. similar the STE primary science fairs.

**Figure 7:**
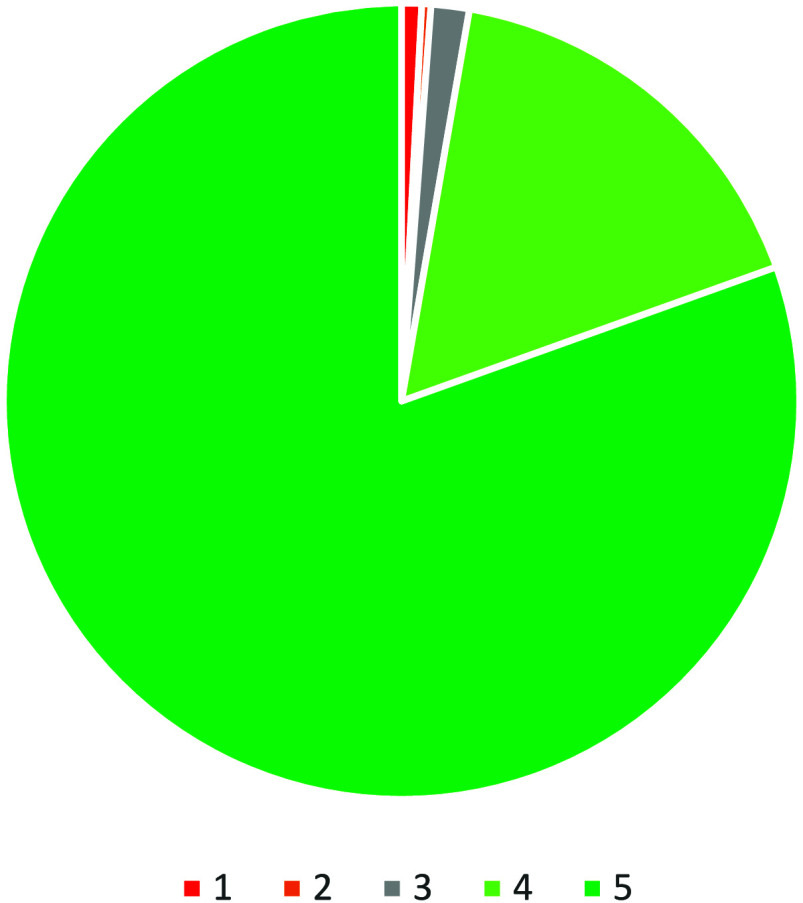
Breakdown of the feedback received for the activity at mixed themed events (*n* = 1352).

The feedback received for MTEs (*n* = 1352, 32% of the total) resulted in a total CSAT score of 97% (Figure 7). This is far above the overall CSAT score of 93% and STE score of 92% (Evaluation Data). Overall, 80% of respondents chose the “I Loved it” (5) response along with 17% who chose “I liked it” (4). Only 1% chose “I hated it” (1), along with 0% choosing “I Did not Like it” (2) and 2% choosing “neutral” (3). In contrast to the STEs, none of the events in this category recorded above 1% for the “I hated it” response (1). With so few negative responses, there are no discernible trends to discuss. Also, since these events occurred at different times, it is impossible to compare the responses by time slots.

However, looking at the MTEs in more depth, it was found that they differed from the STEs in several ways. First, MTEs provided a greater variety of activities and the participants had little or no other science activity to compare against. For both the food festival and the arts festival, the activity described here was the only science activity present. For the agricultural show and STEAM festival, only a small number of science-based activities were present alongside many arts, crafts, food engineering, and technology activities. Noteworthy, for STEs, some of the participants may not be interested in science but are attending the event because others in their family or school are interested. Second, participants may have different expectations for different events. STE participants may have brought with them some expectations for the science activities which may result in some disappointment when the activity does not match those expectations. Finally, the number of participants to Ambassadors at MTEs was consistently lower than STEs as 4:1 in most cases. This gave the participants more time to do the activity and converse with the Ambassadors.

### Further Participant Questions

Further feedback was gathered as a pilot study at a science festival using a digital survey tool. Answers to the questions were a mixture of yes/no, multiple choice and five-point Likert scale (Evaluation Data). From a total of n = 56 responses two groups comprised the largest number of survey respondents, those aged 5–8 (24 respondents) and 9–12 (20 respondents). Consent for participation in the survey from minors was obtained from their parents/guardians which included a review of the questions.

All the survey respondents agreed with the statement “*learning about green energy was fun*”. Notably though, 90% of the 9–12 group strongly agreed (5) while only 67% of the 5–8 group strongly agreed (5). In contrast, 100% of the 13–15 group agreed (4) with none strongly agreeing (5). Interestingly and encouragingly, all respondents answered “yes” to the statement “*I will tell my friends and family about these experiments*”. Also, the 9–12 group were more likely (85%) to strongly agree (5) with the statement “*I liked meeting the scientists*” compared to the 5–8 group (67%).

### Ambassadors Interviews

The Ambassador interviews took place in line with the authors institutional ethics policies. From a total of 41 Ambassadors, 5 individuals volunteered to take part in the online one-to-one interviews. All interviewees expressed that they had a very positive experience, specifically highlighting “*the excitement on the children’s faces when their battery lit up the LED*”. They also enjoyed talking about their research and answering questions. However, they did not like the chaotic and unpredictable nature of running public engagement events in different environments. They also expressed frustration with the salt-water electrolyte, which delayed turnover and distracted them from fully engaging with the participants. One ambassador stated, “*you’d have one.. and everything would work perfectly, and then they could see their friend and it wasn’t working at all*”. Moreover, all the interviewees expressed that the salt-water damaged the skin on their hands over time. The change to playdough was welcomed by all since it meant that they “*could turn over people way faster*”, while also having more time to engage with participants.

All interviewees highlighted the logistical challenges posed by managing the large crowds at STEs, particularly science festivals. One interviewee mentioned that the STEs and MTEs were “*completely different events*”, noting that the audience at MTEs were more varied, consisting of some people who “*were really enthusiastic about science*” and others who “*came because they liked art and crafts*”. Another interviewee noted that the lack of parents/guardians at the primary science fair (STE) meant that groups were generally smaller and more manageable than other events, which was welcomed.

The activity also had a profound impact on the ambassadors’ own view of science. All the interviewees stated that it revived their own enthusiasm for doing science because “*sometimes I think you kind of lose that”*. One interviewee stated that it “*reminds you why you like science*”, which demonstrates the benefits of two-way communication. Another stated that “*it gives me quite a lot of enthusiasm for science again”*, because you can “*forget the broader picture*”. They also expressed a sense of pride that they could act as a role model to “*inspire a younger generation to think that science might be possible for them*” while another interviewee hoped “*that science is more approachable after doing something like this*”. They also hoped that people “*come away realising science is actually for everyone*”. Moreover, they expressed that these activities should help the participants realize “*how everything is related to science, and you can do very cool things with very little materials and very basic stuff*”.

The opportunity to develop various skills were also highlighted. The broad spectrum of people that they encountered meant that they had to “*adapt the activity to the different ages and the different groups*”. This developed their communication skills as they answered hundreds of questions about energy science and their own research. It also helped them develop their leadership and organisational skills while running the activity with different types of people. In general, they expressed that it was a tiring experience, “*but it’s very rewarding*”.

## Conclusions

Presented here is a successful modernisation of the historic voltaic pile for nonformal environments. As one of the first electrical batteries that could provide a continuous current, it is an important milestone in the history of chemistry and is still relevant today as we make greater strides toward green energy. However, the original design lacked the robustness needed for the demands of nonspecialist audiences and nonformal environments. Also, many cells are needed for most applications which can make the experiment tedious and repetitive, especially for nonspecialists. LEDs serve as an efficient and cost-effective application for the voltaic pile, producing observable light from only a few cells.

Playdough was discovered here to be an effective and robust electrolyte through its properties as an ICP. It ensured that the number of cells needed to light an LED remained few, thus reducing the tediousness of the activity. Using the resulting battery to light an LED provided participants with a chemistry application that was not linked to drugs or medicine, and it represented a successful outcome from the activity. This success can help one’s confidence in their scientific abilities by providing a real-world output and confirmation of their experimental skills.

The use of a “Smiley Stand” for gathering feedback about the activity was also successfully deployed here and provided a surprising amount of useful data from many participants. Despite the simplicity, it provided useful trends to aid in the design and preparation of an activity. It was found that busy periods, like lunch time, resulted in a slight increase in negative responses. This indicates that a greater number of Ambassadors are required during these periods to keep the number of participants to Ambassadors low. This can be achieved through real-time data from the Smiley Stand to determine when Ambassadors should take breaks etc.

The theme of “arts and crafts” emerged throughout. The activity here is both science and arts/crafts, representing a mixture of these traditionally siloed concepts. However, this may not be suit some participants, especially those attending STEs since the feedback suggests that the activity was better received by participants at MTEs. However, it is important to note that the feedback received overall was hugely positive, regardless of the event type or time of day. The data here does not suggest that STEs should be avoided, instead, organizers and Ambassadors should plan accordingly for the event type, emphasizing different aspects of an activity at different events. For the activity here, the chemistry should be emphasized at STEs, whereas the arts/crafts skills should be emphasized at MTEs. However, regardless of the event type, more positive feedback was received when the activity was conducted outdoors.

Therefore, scientists should feel confident to engage further with MTE organizers, especially those taking place outdoors, since our experience indicates that these events are very welcoming of science and scientists. Accessible scientific activities add to the diversity of interests catered for at MTEs. As discussed earlier, “Draw A Scientist Tests” (DASTs) have shown that many primary children perceive scientists through outdated stereotypes. It is hoped that the activity format described here can change these stereotypes through meaningful interactions with a diverse range of Ambassadors. Future work will involve studying the expectations of participants for different event types and further exploring the influence of outdoor environments on scientific engagement and enjoyment.

## Supporting Information Available

Activity testing (PDF, DOCX)
Evaluation data (PDF, DOCX)
Worksheets (PDF)
