# Supplementary material for: Do You Want to Make a Battery? Insights from the Development and Evaluation of a Chemistry Public Engagement Activity
Source: J Chem Educ. 2024 Nov 1;101(11):5089–96. doi: 10.1021/acs.jchemed.4c01123 (PMC11562580; doi:10.1021/acs.jchemed.4c01123)
Supplement: Supplementary file 2 — ed4c01123_si_002.docx [file ed4c01123_si_002.docx]

Do you want to make a battery? Insights from the development and evaluation of a chemistry public engagement activity

John O’Donoghue^1*^, Natalia García Doménech^1^, Dearbhla Tully^1^, Niamh McGoldrick^1^, Fiona McArdle^2^, Mary Connolly^2^, Dave J. Otway^3^, Will Daly^3^, Lynette Keeney^4^, and Mervyn Horgan^5^

^1^ School of Chemistry, Trinity College Dublin, Dublin, Ireland, D02 P3X2

^2^ School of Life Sciences, Atlantic Technological University, Sligo, Ireland, F91 YW50

^3^ School of Chemistry, University College Cork, Cork, Ireland, T12 K8AF

^4^ Tyndall National Institute, Cork, Ireland, T12 R5CP

^5^ Lifetime Lab, Old Cork Waterworks, Cork, Ireland, T23 N828

**Activity Testing:**

The effectiveness of Deionized (DI) water, vinegar (typically 5% acetic acid) and dilute NaOH (0.1 M) were first tested as electrolytes for the school workshops. These represented acidic, neutral, and alkaline examples respectively, in addition to their different conductivities. The voltage produced was compared for each electrolyte as the cells were stacked into a pile to create a battery. Although the voltage varied significantly depending on the electrolyte, the current produced remained low, in the order of 200-800 microamps (µA). This limited the real-world applications for these batteries, with low powered Light Emitting Diodes (LEDs) being the most suitable (Table A.4 and A.5).

Overall, Al-Air Battery Equation:

$4Al +3O_{2}+6H_{2}O \to{4 Al(OH)}_{3} (+ 2.71 V)$ (*Eq.1*)

Copper (Cu) Coin Cathode:

$O_{2} +{2 H}_{2}O + {4e}^{-}\to4{OH}^{-} (+0.40 V)$ (*Eq.2*)

$O_{2} +4H^{+} + {4e}^{-}\to2H_{2}O (+1.229 V)$ (*Eq.2a*) (Acid Electrolytes)

Aluminium (Al) Anode:

$Al +3 OH^{-} \to{Al(OH)}_{3} + {3e}^{-} (+2.31 V)$ (*Eq.3*)

Reducing the number of cells needed to light the LED was identified as an important requirement to prevent the activity from becoming tedious and repetitive, in line with previous reports.^1^ The highest voltage was achieved for NaOH, followed by DI water, and vinegar as expected from previous reports.^53^ Alkaline environments can provide a source of OH^−^ ions which can assist in oxidation of the anode (Eq.3).^48^ This increases the effectiveness of the battery and reduces the number of cells required to light the LED (Eq 1 and 2).^53^ The NaOH electrolyte required only 3 cells to light the LED, contrasted with DI Water and Vinegar which required 5 and 6 cells respectively (Table A.1). Although acid solutions can result in different reduction pathways at the cathode (Eq 2a),^53^ they can also interfere with the aluminium oxidization reaction at the anode, so it is undesirable to apply acid electrolytes for Al-air batteries (Eq.3).^53^

Each cell consisted of a copper coin, a cloth ‘spacer’ containing the electrolyte and a disk of aluminium. Historically, cardboard was used as a spacer material and features in other reports of aluminium based Voltaic Piles.^49^ However, here cardboard was found to give inconsistent results for voltage. It also dissolved in the electrolyte over time, significantly reducing its usability. Instead, a felt-based ‘dishcloth’ was found to be the most effective material for retaining the electrolyte, in line with Volta’s experiments and other reports.^44^ The dishcloth is also robust enough to scale the battery for large numbers of cells, functional for a variety of skill levels and is reusable after cleaning. It can also be cut to size, matching that of the coin and the aluminium disks. It also gave more consistent voltage measurements comparing multiple batteries of the same time.

**Adapting for Public Engagement:**

To adapt the activity for public engagement, away from the lab and school environments, first it was decided that 0.1 M NaOH was too hazardous. Vinegar and DI water were also dismissed due to the large number of cells required to light an LED and inconsistencies due to different skill levels. Instead, salt-water (NaCl brine) was employed in line with Volta’s original experiments.^44^ From testing, salt-water required about 4 cells to light an LED, which represented a comprise compared to the 3 needed for dilute NaOH. Also, like aluminium, table salt is food grade, easily recognisable as an everyday item and is not generally considered a lab chemical. Before gathering feedback, the salt-water version of the activity was tested at 2 Science Festivals to establish format and logistics.

Although the salt-water electrolyte only needed 4 cells to light the LED, a larger number of cells ensured success for all participants. However, from the pilot events it was discovered that the copper coins used as the cathode are in fact copper coated steel cores. After only a few uses, the thin copper coating oxidises and erodes due to corrosion caused by the salt-water (brine) electrolyte. This impacted the effectiveness of the battery as time went on, occasionally requiring more than 8 cells to light the LED. Initially, cleaning the coins with vinegar and/or fresh water was somewhat successful, but it became increasingly difficult for the Ambassadors to maintain an enjoyable experience for the participants. As a result, a large supply of coins was needed, LEDs were consumed throughout the day and the activity ‘failed’ for some participants, especially during busy periods.

Therefore, an intrinsically conducting polymer (ICP) electrolyte was employed instead in the form of ‘playdough’ (commercial name Play-Doh) which produced similar voltage and functionality as salt-water. Noteworthy, it was also discovered here that the amylose gel extraction can serve as a functional ICP on its own, without the addition of salt or other additives. However, the lower conductivity of the amylose gel due to the lack of salt is evident with reduced voltages (Table A.1). It also takes a minimum of 4 cells for the amylose gel to light up the LED compared to 3 or 4 cells for the salt containing playdough. It was also noted that the dyes used in commercial playdough may also contribute to conductivity, with different voltages obtained for different colours (Table A.2 and A3). Further discussion in relation to ICP electrolytes is beyond the scope of this report and is currently the subject of further work.

**Voltage data collected during electrolyte testing:**

Table A.1: Different electrolytes held in place with a cloth spacer between commercially available aluminium foil and copper coated coins. Highlighted in green is the first indication of the LED lighting.

| Number of cells | 0.1M NaOH Voltage | DI Water  Voltage | Vinegar  (5% Acetic Acid)  Voltage | Salt-Water (Brine)  Voltage | Homemade Playdough Voltage | Amylose Gel  Voltage |
| --- | --- | --- | --- | --- | --- | --- |
| 1 | 1.10 | 0.66 | 0.54 | 0.65 | 0.67 | 0.71 |
| 2 | 1.89 | 1.22 | 0.89 | 1.26 | 1.23 | 1.19 |
| 3 | 2.76 | 1.88 | 1.31 | 1.87 | 1.91 | 1.57 |
| 4 | 3.81 | 2.46 | 1.85 | 2.49 | 2.59 | 2.24 |
| 5 | 4.77 | 2.88 | 2.56 | 3.05 | 3.25 | 2.72 |
| 6 | 5.83 | 3.42 | 2.98 | 3.59 | 3.84 | 3.31 |
| 7 | 6.75 | 3.98 | 3.53 | 4.20 | 4.50 | 3.92 |
| 8 | 7.58 | 4.43 | 4.05 | 4.74 | 5.18 | 4.53 |
| 9 | 8.76 | 4.73 | 4.71 | 5.35 | 5.70 | 4.89 |
| 10 | 9.69 | 5.35 | 5.33 | 5.87 | 6.38 | 5.37 |

Table A.2: Different colours of commercial playdough sandwiched between zinc coated (galvanised) washers and copper coated coins. Highlighted in green is the first indication of the LED lighting.

| Number of cells | Pink 1  Voltage | Pink 2  Voltage | Blue 1  Voltage | Blue 2  Voltage | Green 1  Voltage | Green 2  Voltage | Black  Voltage | White  Voltage |
| --- | --- | --- | --- | --- | --- | --- | --- | --- |
| 1 | 0.80 | 0.79 | 0.72 | 0.74 | 0.75 | 0.76 | 0.73 | 0.73 |
| 2 | 1.60 | 1.57 | 1.55 | 1.48 | 1.49 | 1.56 | 1.52 | 1.46 |
| 3 | 2.40 | 2.33 | 2.24 | 2.22 | 2.24 | 2.34 | 2.25 | 2.19 |
| 4 | 3.17 | 3.11 | 2.99 | 2.97 | 2.96 | 3.11 | 2.97 | 2.88 |
| 5 | 3.96 | 3.85 | 3.76 | 3.73 | 3.78 | 3.89 | 3.71 | 3.64 |
| 6 | 4.68 | 4.60 | 4.45 | 4.43 | 4.53 | 4.60 | 4.43 | 4.36 |
| 7 | 5.48 | 5.33 | 5.10 | 5.14 | 5.27 | 5.33 | 5.13 | 5.05 |
| 8 | 6.30 | 6.07 | 5.87 | 5.85 | 6.01 | 6.04 | 5.84 | 5.71 |
| 9 | 7.06 | 6.83 | 6.61 | 6.55 | 6.73 | 6.81 | 6.57 | 6.37 |
| 10 | 7.79 | 7.47 | 7.27 | 7.22 | 7.40 | 7.50 | 7.22 | 7.01 |

Table A.3: Different colours of commercial playdough sandwiched between commercially available aluminium foil and copper coated coins. Highlighted in green is the first indication of the LED lighting.

| Number of cells | Pink 1  Voltage | Pink 2  Voltage | Blue 1  Voltage | Blue 2  Voltage | Green 1  Voltage | G2  Voltage | Black  Voltage | White  Voltage |
| --- | --- | --- | --- | --- | --- | --- | --- | --- |
| 1 | 0.70 | 0.68 | 0.65 | 0.63 | 0.65 | 0.71 | 0.69 | 0.76 |
| 2 | 1.29 | 1.30 | 1.25 | 1.21 | 1.22 | 1.29 | 1.26 | 1.31 |
| 3 | 1.84 | 1.93 | 1.87 | 1.71 | 1.84 | 1.91 | 1.94 | 1.90 |
| 4 | 2.43 | 2.52 | 2.49 | 2.37 | 2.34 | 2.58 | 2.58 | 2.54 |
| 5 | 3.03 | 3.16 | 3.04 | 2.95 | 2.96 | 3.21 | 3.22 | 3.16 |
| 6 | 3.53 | 3.81 | 3.65 | 3.53 | 3.56 | 3.78 | 3.79 | 3.71 |
| 7 | 4.08 | 4.33 | 4.24 | 4.06 | 4.14 | 4.46 | 4.40 | 4.31 |
| 8 | 4.69 | 4.99 | 4.79 | 4.16 | 4.66 | 5.08 | 4.94 | 4.92 |
| 9 | 5.20 | 5.51 | 5.35 | 4.80 | 5.25 | 5.64 | 5.60 | 5.56 |
| 10 | 5.83 | 6.07 | 5.93 | 5.35 | 5.85 | 6.21 | 6.24 | 6.13 |

**LED Data (Supplied by manufacturer):**

**Table A.4: Absolute Maximum Ratings (TA = 25°C)**

| **Items** | **Symbol** | **Absolute Maximum Rating** | **Unit** |
| --- | --- | --- | --- |
| **Forward Current** | I_F_ | 30 | mA |
| **Peak Forward Current** | I_FP_ | 100 | mA |
| **Reverse Voltage** | V_R_ | 5 | V |
| **Power Dissipation** | P_D_ | 120 | mW |
| **Operation Temperature** | T_opr_ | -40 ~ +95 | °C |
| **Storage Temperature** | T_stg_ | -40 ~ +100 | °C |

**Table A.5 Typical Electrical and Optical Characteristics (T_A_ 25°C)**

| **Characteristics** | **Colour** | **Symbol** | **Condition** | **Unit** | **Minimum** | **Typical** | **Maximum** |
| --- | --- | --- | --- | --- | --- | --- | --- |
| Forward Voltage | Blue/Green | V_F_ | IF = 20 mA | V |  | 3.20 | 4.00 |
| Reverse Current | Blue/Green | I_R_ | VR = 5 V | μA |  |  | 100 |
| Dominant Wavelength | Blue | λ_D_ | IF = 20 mA | nm | 465 | 470 | 480 |
|  | Green | λ_D_ | IF = 20 mA | nm | 520 | 527 | 535 |
| Luminous Intensity | Blue | I_V_ | IF = 20 mA | mcd | 4180 | 11000 |  |
|  | Green | I_V_ | IF = 20 mA | mcd | 16800 | 38000 |  |
