# Supplementary material for: Do You Want to Make a Battery? Insights from the Development and Evaluation of a Chemistry Public Engagement Activity
Source: J Chem Educ. 2024 Nov 1;101(11):5089–96. doi: 10.1021/acs.jchemed.4c01123 (PMC11562580; doi:10.1021/acs.jchemed.4c01123)
Supplement: Supplementary file 3 — ed4c01123_si_003.pdf [file ed4c01123_si_003.pdf]

Do you want to make a battery? Insights from the development and evaluation of a chemistry public engagement activity

John O'Donoghue[1*], Natalia García Doménech[1], Dearbhla Tully[1], Niamh McGoldrick[1], Fiona McArdle[2], Mary Connolly[2], Dave J. Otway[3], Will Daly[3], Lynette Keeney[4], and Mervyn Horgan[5]

[1] School of Chemistry, Trinity College Dublin, Dublin, Ireland, D02 P3X2

[2] School of Life Sciences, Atlantic Technological University, Sligo, Ireland, F91 YW50

[3] School of Chemistry, University College Cork, Cork, Ireland, T12 K8AF

[4] Tyndall National Institute, Cork, Ireland, T12 R5CP

[5] Lifetime Lab, Old Cork Waterworks, Cork, Ireland, T23 N828

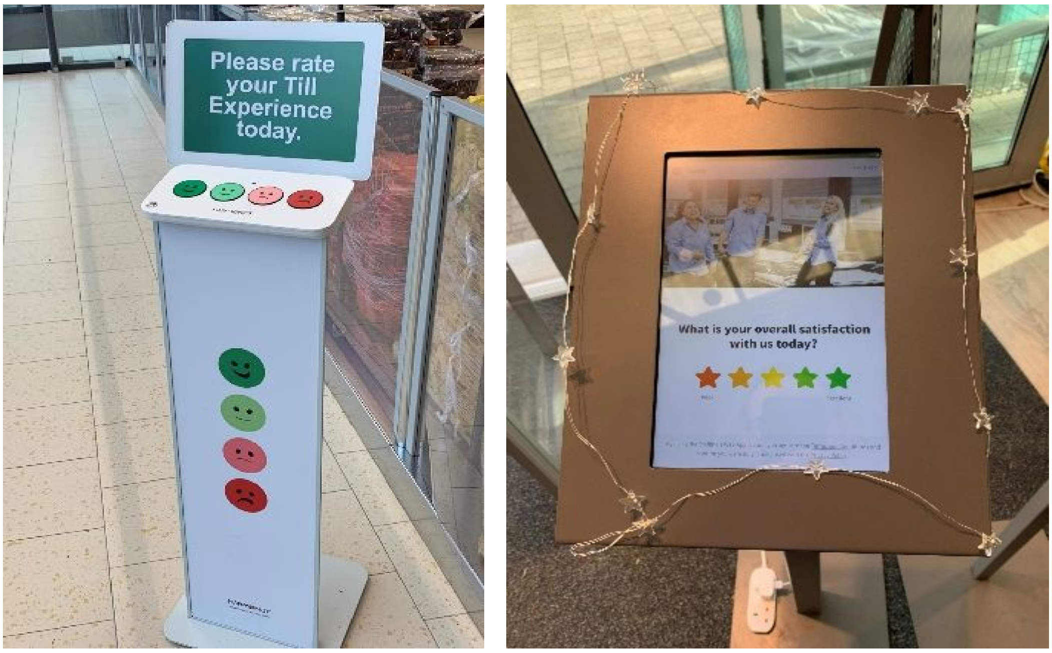

Figure B1: Examples of other 'Smiley Stands' used by retailers such as supermarkets to gather feedback from customers.

Ambassador Interviews Prompt Questions:

1. Please describe your personal impressions of doing these experiences overall.
2. Please outline any differences that you noticed between each of the events that you were involved with?
3. Do you think these experiences had an impact on you, if so, please provide some details.
4. What would you say is your favourite and your least favourite parts of doing these activities?
5. What impact, if any, do you think this activity had on the people you engaged with?

Participant Feedback from Smiley Stand

Table B.1: Total feedback responses per event

Activity Type	Event	Total	Likert 1	Likert 2	Likert 3	Likert 4	Likert 5	CSAT
Salt-Water	Agricultural Event 2022	204	3	0	2	20	179	98%
Salt-Water	Science Festival 2022	158	4	2	7	35	110	92%
Salt-Water	Primary Science Fair 2023	672	22	3	20	104	523	93%
Playdough	Science Festival 2023	422	13	7	16	90	296	91%
Playdough	STEAM Festival 2023	303	2	0	3	57	241	98%
Playdough	Primary Science Fair 2024	931	54	11	40	212	614	89%
Playdough	Science Festival 2024	742	13	8	24	160	537	94%
Playdough	Food Festival 2024	142	0	0	4	24	114	97%
Playdough	Arts Festival 2024	92	1	1	1	15	74	97%
Playdough	STEAM Festival 2024	611	5	4	11	111	480	97%
All	Totals	4227	117	36	128	828	3168	-
All	Percentages	100%	3%	1%	3%	19%	74%	93%

Table B.2: Total feedback from Science Themed Events by hour

Time	Total	Likert 1	Likert 2	Likert 3	Likert 4	Likert 5
**9:00-9:59 am**	70	1	0	1	15	53
**10:00-10:59 am**	295	11	3	12	54	215
**11:00-11:59 am**	592	24	5	19	136	408
**12:00-12:59 pm**	663	35	6	23	120	479
**1:00-1:59 pm**	537	16	4	26	106	385
**2:00-2:59 pm**	303	9	2	14	59	219
**3:00-3:59 pm**	217	4	5	9	55	144
**4:00-4:59 pm**	177	3	3	2	45	124
**5:00-5:59 pm**	71	3	3	1	11	53
**Total**	2925	106	31	107	601	2080
**Percentage**	100%	4%	1%	4%	21%	71%

Table B.3: Total Feedback from Mixed Themed Events by hour

Mixed Themed Events Overall	Total	Likert 1	Likert 2	Likert 3	Likert 4	Likert 5
**9:00-9:59 am**	7	0	0	0	1	6
**10:00-10:59 am**	93	1	0	0	9	83
**11:00-11:59 am**	168	1	2	2	30	133
**12:00-12:59 pm**	266	0	0	2	39	225
**1:00-1:59 pm**	225	3	2	5	36	179
**2:00-2:59 pm**	201	2	1	2	39	157
**3:00-3:59 pm**	259	4	0	7	50	198
**4:00-4:59 pm**	122	0	0	3	21	98
**5:00-5:59 pm**	11	0	0	0	2	9
**Total**	1352	11	5	21	227	1088
**Percentage**	100%	1%	0%	2%	17%	80%

Detailed Pilot Survey:

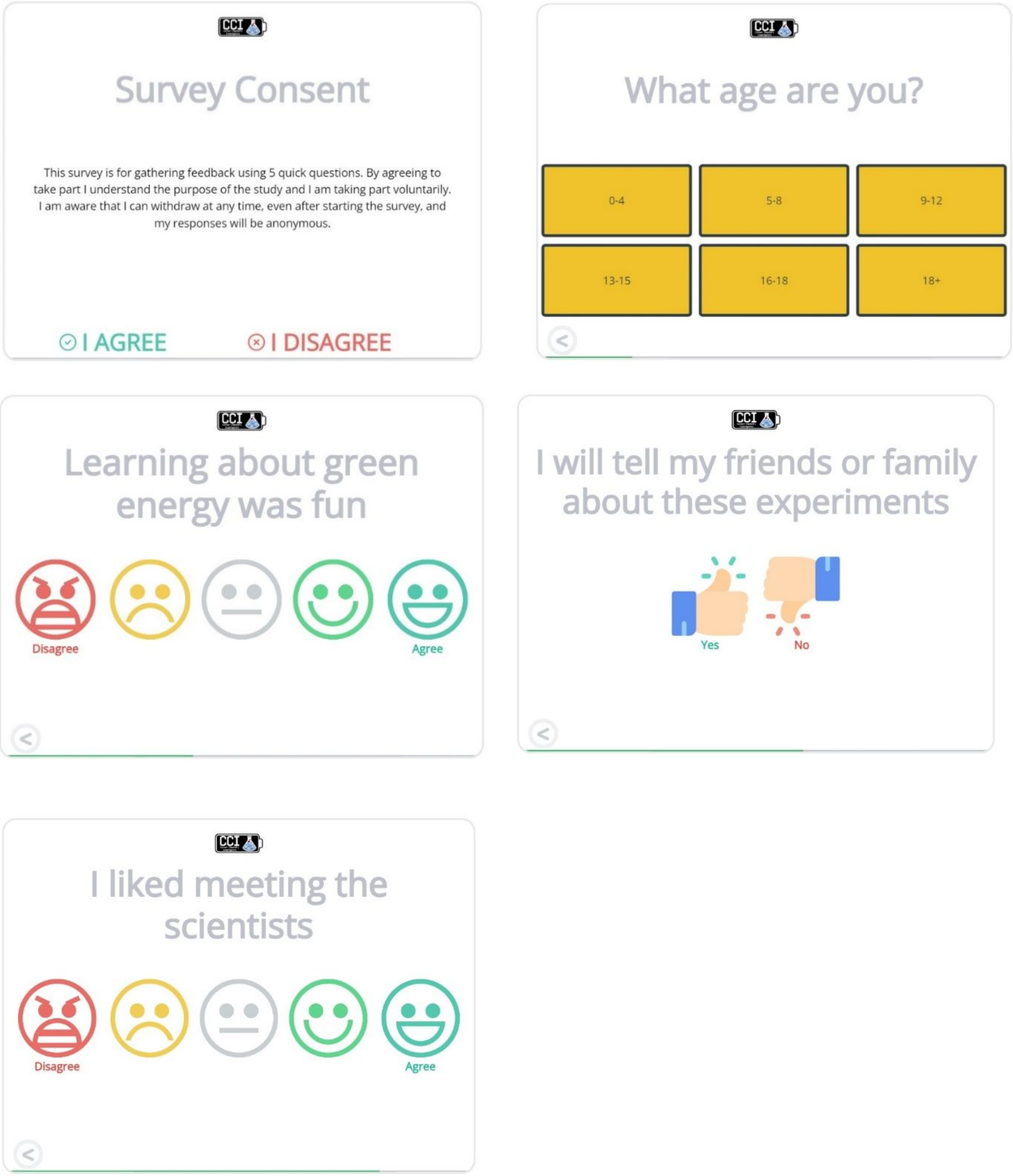

Figure B2: Examples of the questions and visuals used for the detailed pilot study.

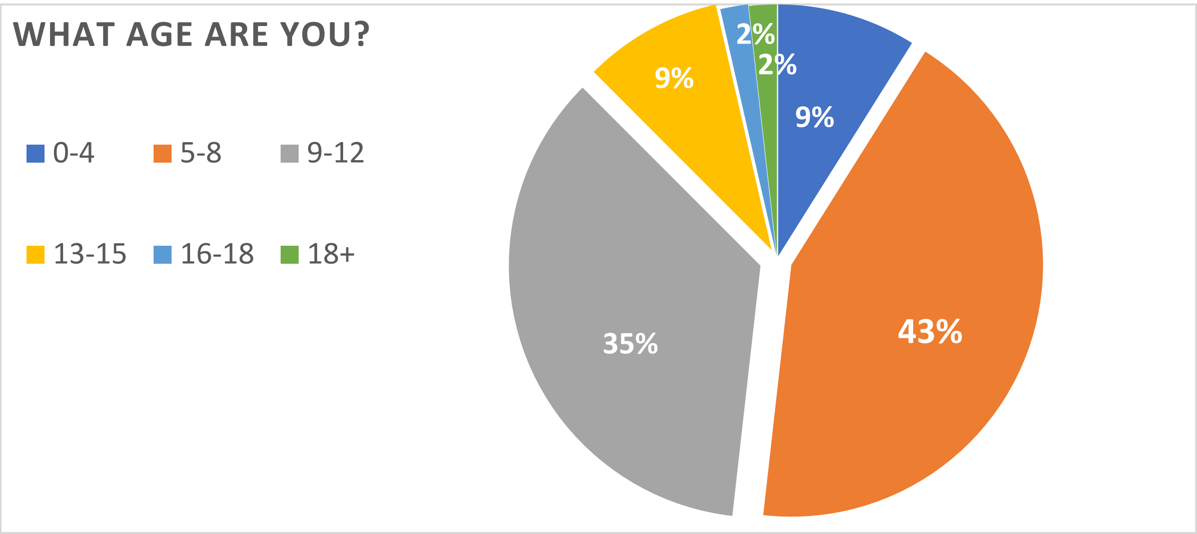

Figure B3: Breakdown of respondents by age for detailed pilot study.

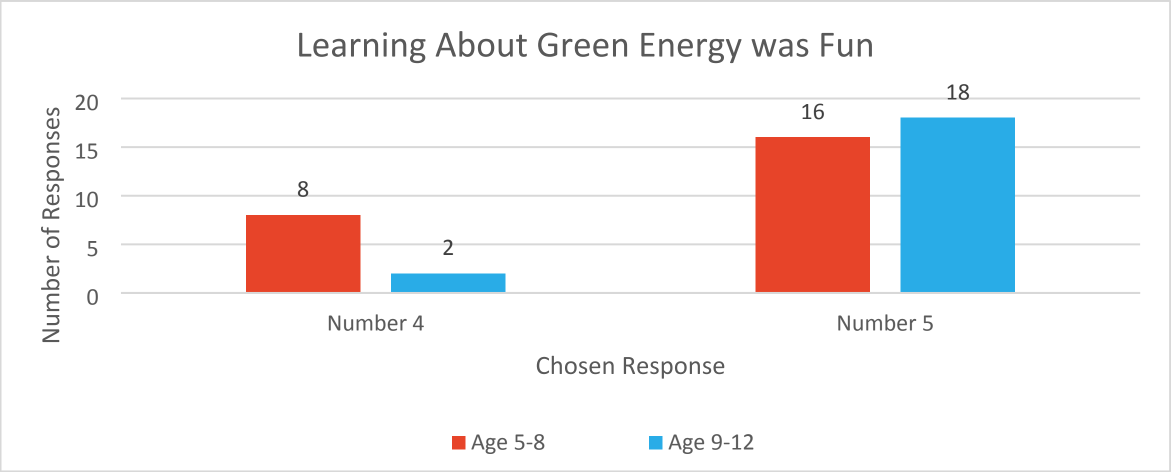

Figure B4: A comparison of responses for the question "*Learning about Green Energy was fun*" for the two largest cohorts of respondents

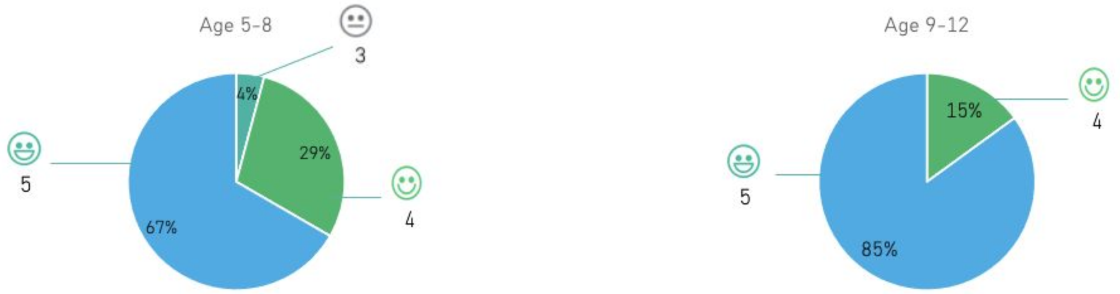

Figure B5: A comparison of responses for the question "*I Liked Meeting the Scientists*" for the two largest cohorts of respondents.
