# Supplementary material for: Do You Want to Make a Battery? Insights from the Development and Evaluation of a Chemistry Public Engagement Activity
Source: J Chem Educ. 2024 Nov 1;101(11):5089–96. doi: 10.1021/acs.jchemed.4c01123 (PMC11562580; doi:10.1021/acs.jchemed.4c01123)
Supplement: Supplementary file 4 — ed4c01123_si_004.docx [file ed4c01123_si_004.docx]

Do you want to make a battery? Insights from the development and evaluation of a chemistry public engagement activity

John O’Donoghue^1*^, Natalia García Doménech^1^, Dearbhla Tully^1^, Niamh McGoldrick^1^, Fiona McArdle^2^, Mary Connolly^2^, Dave J. Otway^3^, Will Daly^3^, Lynette Keeney^4^, and Mervyn Horgan^5^

^1^ School of Chemistry, Trinity College Dublin, Dublin, Ireland, D02 P3X2

^2^ School of Life Sciences, Atlantic Technological University, Sligo, Ireland, F91 YW50

^3^ School of Chemistry, University College Cork, Cork, Ireland, T12 K8AF

^4^ Tyndall National Institute, Cork, Ireland, T12 R5CP

^5^ Lifetime Lab, Old Cork Waterworks, Cork, Ireland, T23 N828


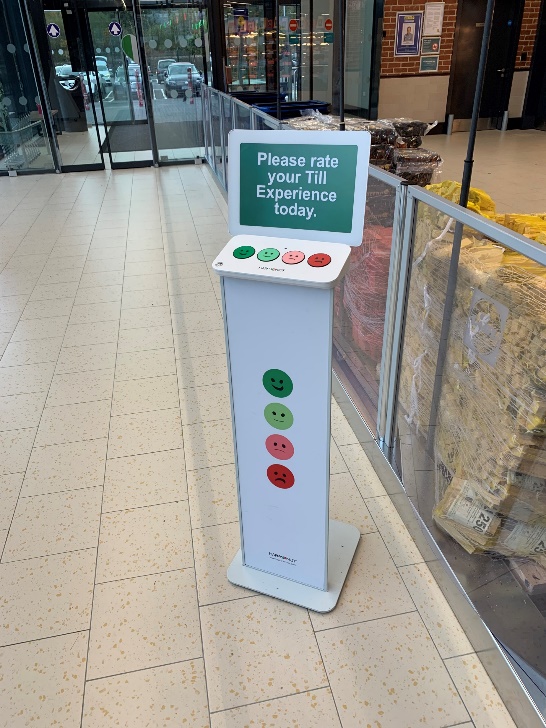

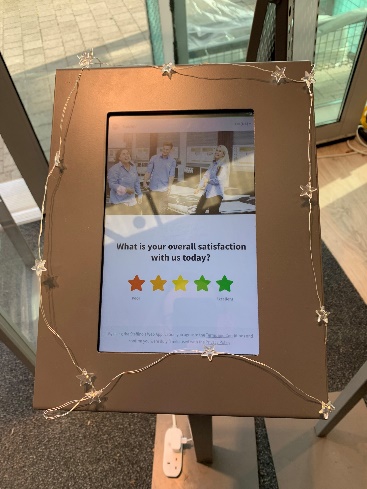


Figure B1: Examples of other ‘Smiley Stands’ used by retailers such as supermarkets to gather feedback from customers.

**Ambassador Interviews Prompt Questions:**

1. Please describe your personal impressions of doing these experiences overall.
2. Please outline any differences that you noticed between each of the events that you were involved with?
3. Do you think these experiences had an impact on you, if so, please provide some details.
4. What would you say is your favourite and your least favourite parts of doing these activities?
5. What impact, if any, do you think this activity had on the people you engaged with?

**Participant Feedback from Smiley Stand**

Table B.1: Total feedback responses per event

| Activity Type | Event | Total | Likert 1 | Likert 2 | Likert 3 | Likert 4 | Likert 5 | CSAT |
| --- | --- | --- | --- | --- | --- | --- | --- | --- |
| Salt-Water | Agricultural Event 2022 | 204 | 3 | 0 | 2 | 20 | 179 | 98% |
| Salt-Water | Science Festival 2022 | 158 | 4 | 2 | 7 | 35 | 110 | 92% |
| Salt-Water | Primary Science Fair 2023 | 672 | 22 | 3 | 20 | 104 | 523 | 93% |
| Playdough | Science Festival 2023 | 422 | 13 | 7 | 16 | 90 | 296 | 91% |
| Playdough | STEAM Festival 2023 | 303 | 2 | 0 | 3 | 57 | 241 | 98% |
| Playdough | Primary Science Fair 2024 | 931 | 54 | 11 | 40 | 212 | 614 | 89% |
| Playdough | Science Festival 2024 | 742 | 13 | 8 | 24 | 160 | 537 | 94% |
| Playdough | Food Festival 2024 | 142 | 0 | 0 | 4 | 24 | 114 | 97% |
| Playdough | Arts Festival 2024 | 92 | 1 | 1 | 1 | 15 | 74 | 97% |
| Playdough | STEAM Festival 2024 | 611 | 5 | 4 | 11 | 111 | 480 | 97% |
| All | Totals | 4227 | 117 | 36 | 128 | 828 | 3168 | - |
| All | Percentages | 100% | 3% | 1% | 3% | 19% | 74% | 93% |

Table B.2: Total feedback from Science Themed Events by hour

| **Time** | **Total** | **Likert 1** | **Likert 2** | **Likert 3** | **Likert 4** | **Likert 5** |
| --- | --- | --- | --- | --- | --- | --- |
| **9:00-9:59 am** | 70 | 1 | 0 | 1 | 15 | 53 |
| **10:00-10:59 am** | 295 | 11 | 3 | 12 | 54 | 215 |
| **11:00-11:59 am** | 592 | 24 | 5 | 19 | 136 | 408 |
| **12:00-12:59 pm** | 663 | 35 | 6 | 23 | 120 | 479 |
| **1:00-1:59 pm** | 537 | 16 | 4 | 26 | 106 | 385 |
| **2:00-2:59 pm** | 303 | 9 | 2 | 14 | 59 | 219 |
| **3:00-3:59 pm** | 217 | 4 | 5 | 9 | 55 | 144 |
| **4:00-4:59 pm** | 177 | 3 | 3 | 2 | 45 | 124 |
| **5:00-5:59 pm** | 71 | 3 | 3 | 1 | 11 | 53 |
| **Total** | 2925 | 106 | 31 | 107 | 601 | 2080 |
| **Percentage** | 100% | 4% | 1% | 4% | 21% | 71% |

Table B.3: Total Feedback from Mixed Themed Events by hour

| **Mixed Themed Events Overall** | **Total** | **Likert 1** | **Likert 2** | **Likert 3** | **Likert 4** | **Likert 5** |
| --- | --- | --- | --- | --- | --- | --- |
| **9:00-9:59 am** | 7 | 0 | 0 | 0 | 1 | 6 |
| **10:00-10:59 am** | 93 | 1 | 0 | 0 | 9 | 83 |
| **11:00-11:59 am** | 168 | 1 | 2 | 2 | 30 | 133 |
| **12:00-12:59 pm** | 266 | 0 | 0 | 2 | 39 | 225 |
| **1:00-1:59 pm** | 225 | 3 | 2 | 5 | 36 | 179 |
| **2:00-2:59 pm** | 201 | 2 | 1 | 2 | 39 | 157 |
| **3:00-3:59 pm** | 259 | 4 | 0 | 7 | 50 | 198 |
| **4:00-4:59 pm** | 122 | 0 | 0 | 3 | 21 | 98 |
| **5:00-5:59 pm** | 11 | 0 | 0 | 0 | 2 | 9 |
| **Total** | 1352 | 11 | 5 | 21 | 227 | 1088 |
| **Percentage** | 100% | 1% | 0% | 2% | 17% | 80% |


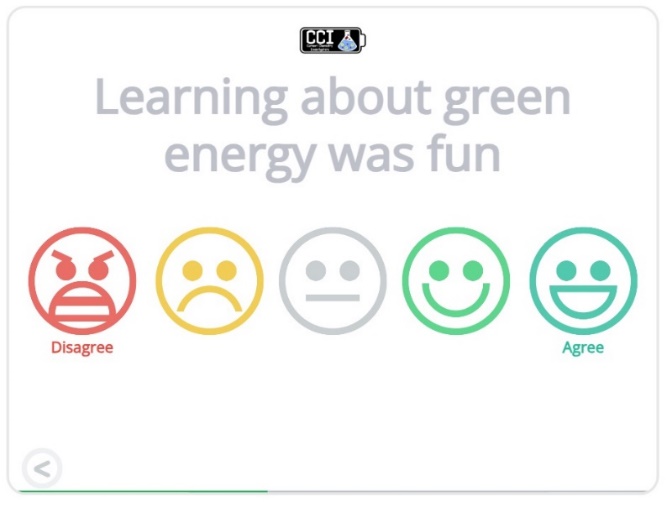

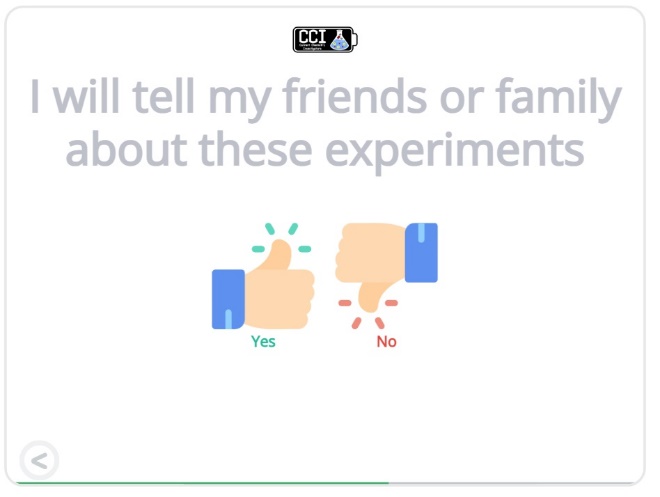

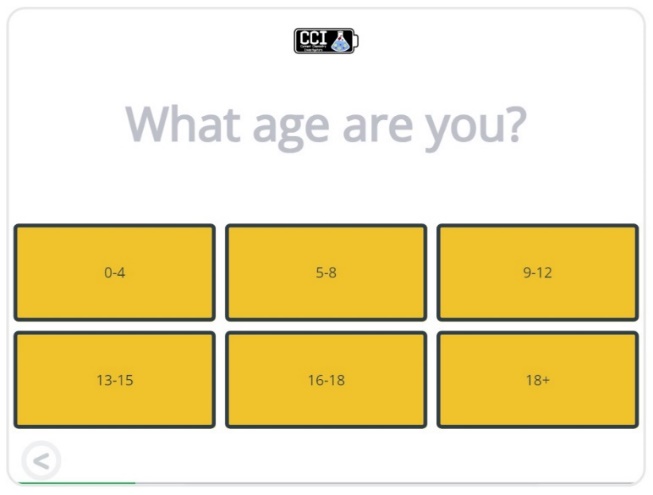

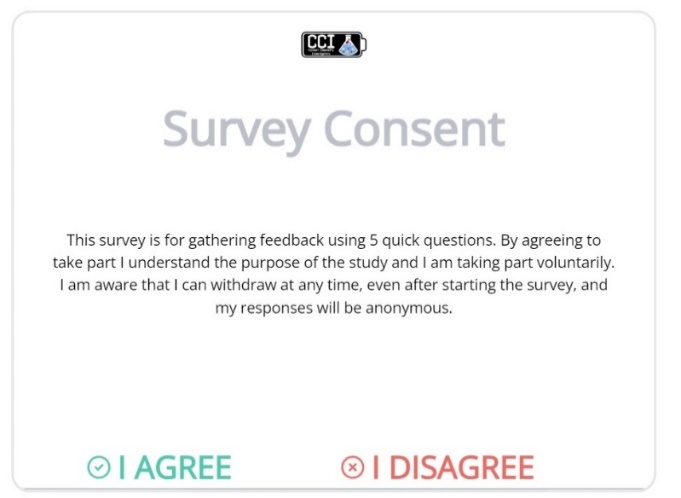
**Detailed Pilot Survey:**


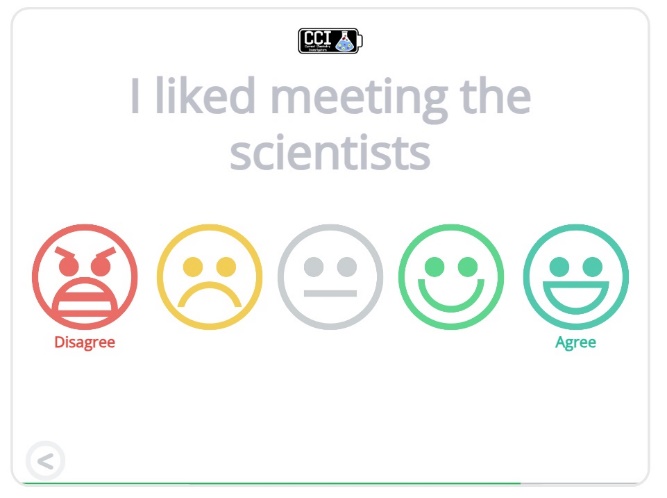


Figure B2: Examples of the questions and visuals used for the detailed pilot study.

Figure B3: Breakdown of respondents by age for detailed pilot study.

Figure B4: A comparison of responses for the question “*Learning about Green Energy was fun*” for the two largest cohorts of respondents

**
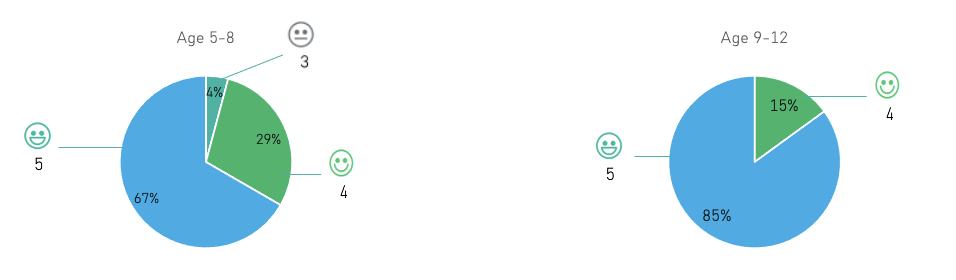
**

Figure B5: A comparison of responses for the question “*I Liked Meeting the Scientists*” for the two largest cohorts of respondents.
