# Supplementary material for: Do You Want to Make a Battery? Insights from the Development and Evaluation of a Chemistry Public Engagement Activity
Source: J Chem Educ. 2024 Nov 1;101(11):5089–96. doi: 10.1021/acs.jchemed.4c01123 (PMC11562580; doi:10.1021/acs.jchemed.4c01123)
Supplement: Supplementary file 5 — ed4c01123_si_005.pdf [file ed4c01123_si_005.pdf]

Al-Air Battery

The Aluminium foil will oxidise into Al ions. The electrolyte (Play-Doh) helps to move the charge. The coin acts as an inert conductor for the oxygen reduction reaction to complete the circuit. Play-Doh contains a lot of salt, which helps move the charge.

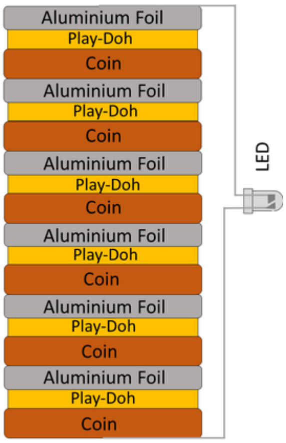

- **Electricity:** is a flow of electric charge, carried by charged ions and electrons
- **Cell:** a device capable of generating electrical energy from a chemical reaction.
- **Battery:** several cells connected that store chemical energy for conversion to electricity.

Science Foundation Ireland sfi For what's next · Trinity College Dublin The University of Dublin · ATU · Lifetime Lab · Tyndall National Institute · UCC

Al-Air Battery

The Aluminium foil will oxidise into Al ions. The electrolyte (Play-Doh) helps to move the charge. The coin acts as an inert conductor for the oxygen reduction reaction to complete the circuit. Play-Doh contains a lot of salt, which helps move the charge.

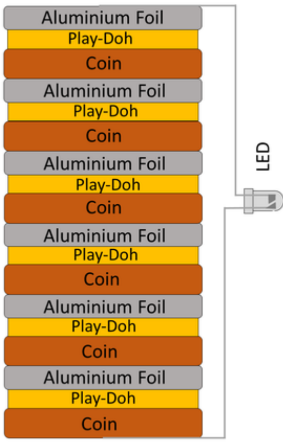

- **Electricity:** is a flow of electric charge, carried by charged ions and electrons
- **Cell:** a device capable of generating electrical energy from a chemical reaction.
- **Battery:** several cells connected that store chemical energy for conversion to electricity.

Al-Air Battery

The Aluminium foil will oxidise into Al ions. The electrolyte (Play-Doh) helps to move the charge. The coin acts as an inert conductor for the oxygen reduction reaction to complete the circuit. Play-Doh contains a lot of salt, which helps move the charge.

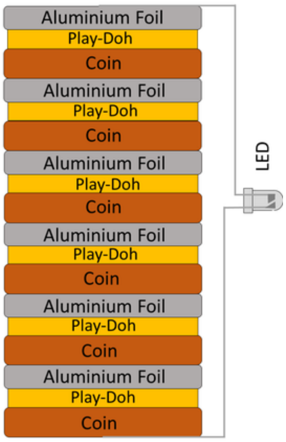

- **Electricity:** is a flow of electric charge, carried by charged ions and electrons
- **Cell:** a device capable of generating electrical energy from a chemical reaction.
- **Battery:** several cells connected that store chemical energy for conversion to electricity.

Al-Air Battery

The Aluminium foil will oxidise into Al ions. The electrolyte (Play-Doh) helps to move the charge. The coin acts as an inert conductor for the oxygen reduction reaction to complete the circuit. Play-Doh contains a lot of salt, which helps move the charge.

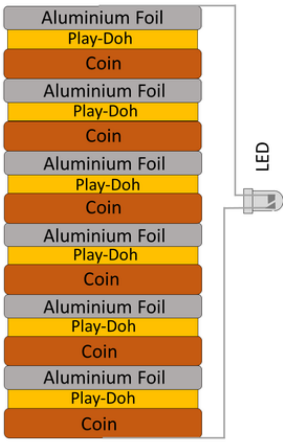

- **Electricity:** is a flow of electric charge, carried by charged ions and electrons
- **Cell:** a device capable of generating electrical energy from a chemical reaction.
- **Battery:** several cells connected that store chemical energy for conversion to electricity.

Al-Air Battery

The Aluminium foil will oxidise into Al ions. The electrolyte (Play-Doh) helps to move the charge. The coin acts as an inert conductor for the oxygen reduction reaction to complete the circuit. Play-Doh contains a lot of salt, which helps move the charge.

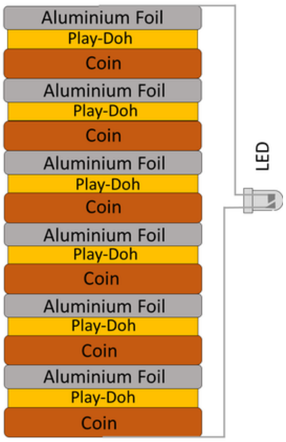

- **Electricity:** is a flow of electric charge, carried by charged ions and electrons
- **Cell:** a device capable of generating electrical energy from a chemical reaction.
- **Battery:** several cells connected that store chemical energy for conversion to electricity.

Al-Air Battery

The Aluminium foil will oxidise into Al ions. The electrolyte (Play-Doh) helps to move the charge. The coin acts as an inert conductor for the oxygen reduction reaction to complete the circuit. Play-Doh contains a lot of salt, which helps move the charge.

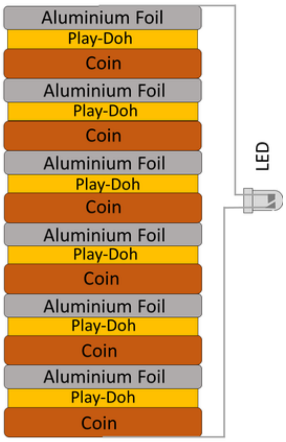

- **Electricity:** is a flow of electric charge, carried by charged ions and electrons
- **Cell:** a device capable of generating electrical energy from a chemical reaction.
- **Battery:** several cells connected that store chemical energy for conversion to electricity.

CCI
Current Chemistry Investigators

1. Cut out 6 disks of aluminium foil -Use the coins as a template and cut inside of the line.
2. Create 6 small balls of play-doh
3. Stack the coin, play-doh & aluminium in the correct order to create a battery.
4. Connect the **LONG** leg of the LED to the coin, and the **SHORT** leg to the foil.
5. Keep adding cells until the LED lights up, how many cells does it take?

More info on the activity, research and ethics at:

www.currentchemistryinvestigators.com

CCI
Current Chemistry Investigators

1. Cut out 6 disks of aluminium foil -Use the coins as a template and cut inside of the line.
2. Create 6 small balls of play-doh
3. Stack the coin, play-doh & aluminium in the correct order to create a battery.
4. Connect the **LONG** leg of the LED to the coin, and the **SHORT** leg to the foil.
5. Keep adding cells until the LED lights up, how many cells does it take?

More info on the activity, research and ethics at:

www.currentchemistryinvestigators.com

CCI
Current Chemistry Investigators

1. Cut out 6 disks of aluminium foil -Use the coins as a template and cut inside of the line.
2. Create 6 small balls of play-doh
3. Stack the coin, play-doh & aluminium in the correct order to create a battery.
4. Connect the **LONG** leg of the LED to the coin, and the **SHORT** leg to the foil.
5. Keep adding cells until the LED lights up, how many cells does it take?

More info on the activity, research and ethics at:

www.currentchemistryinvestigators.com

CCI
Current Chemistry Investigators

1. Cut out 6 disks of aluminium foil -Use the coins as a template and cut inside of the line.
2. Create 6 small balls of play-doh
3. Stack the coin, play-doh & aluminium in the correct order to create a battery.
4. Connect the **LONG** leg of the LED to the coin, and the **SHORT** leg to the foil.
5. Keep adding cells until the LED lights up, how many cells does it take?

More info on the activity, research and ethics at:

www.currentchemistryinvestigators.com

CCI
Current Chemistry Investigators

1. Cut out 6 disks of aluminium foil -Use the coins as a template and cut inside of the line.
2. Create 6 small balls of play-doh
3. Stack the coin, play-doh & aluminium in the correct order to create a battery.
4. Connect the **LONG** leg of the LED to the coin, and the **SHORT** leg to the foil.
5. Keep adding cells until the LED lights up, how many cells does it take?

More info on the activity, research and ethics at:

www.currentchemistryinvestigators.com

CCI
Current Chemistry Investigators

1. Cut out 6 disks of aluminium foil -Use the coins as a template and cut inside of the line.
2. Create 6 small balls of play-doh
3. Stack the coin, play-doh & aluminium in the correct order to create a battery.
4. Connect the **LONG** leg of the LED to the coin, and the **SHORT** leg to the foil.
5. Keep adding cells until the LED lights up, how many cells does it take?

More info on the activity, research and ethics at:

www.currentchemistryinvestigators.com

Battery Investigators

- **Electricity** is a flow of charged **electrons**.
- **Cell**: a device capable of generating electrical energy from a chemical reaction.
- **Battery**: several cells connected together that store chemical energy for later conversion to electrical energy.

Chemical Reactions

Oxidation at the Aluminium Foil electrode:

Reduction at the Inert Coin electrode:

Overall Balanced Equation:

Al–Air Battery

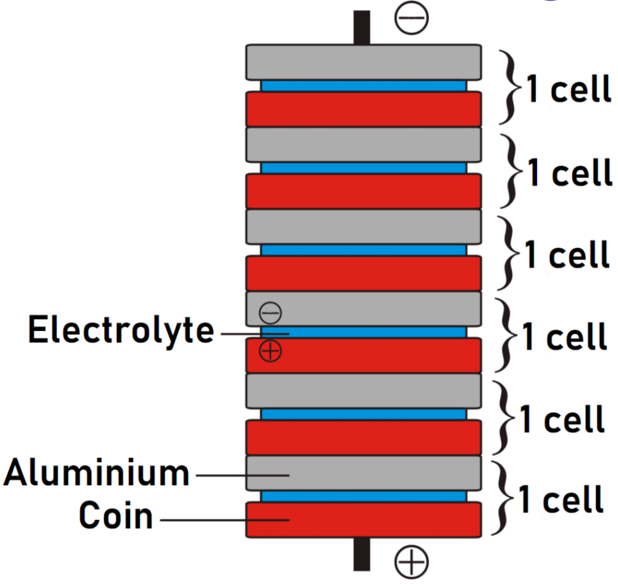

Trinity College Dublin
Coláiste na Tríonóide, Baile Átha Cliath
The University of Dublin

Instructions

sfi
Fondúireacht Eolaíochta Éireann
Dá bhfuil romhainn
Science Foundation Ireland
For what's next

- Cut out the aluminium foil and cloth disks.
- Use the 5c coin as a template – cut inside of the line that you draw.
- Wet cloth in the electrolyte – it should be damp, not soaking!
- Place cloth on top of the coin and cover with foil (**first cell**).
- Measure the voltage produced between the aluminium and coin.
- Make a **second cell** and place it on top of **first cell**.
- Measure the voltage of the **2 cell** battery you just made.
- Repeat this process until you have a battery with **6 cells**.

Cell Number	Water (Volts)	Acetic Acid (Vinegar) (Volts)	NaOH (Volts)
1			
2			
3			
4			
5			
6			

1. Fill table in with volts.
2. Plot a graph with all electrolytes.
3. Which electrolyte works best?
4. Least number of cells to light the LED?

Best electrolyte:	Cells for LED:

Cell number on the X-Axis
Voltage on the Y-Axis
